# Contribution of the EnvZ/OmpR two-component system to growth, virulence and stress tolerance of colistin-resistant *Aeromonas hydrophila*

**DOI:** 10.3389/fmicb.2022.1032969

**Published:** 2022-10-12

**Authors:** Gang Xiao, Xiaofeng Zheng, Jiyun Li, Yang Yang, Jie Yang, Ning Xiao, Junqi Liu, Zhiliang Sun

**Affiliations:** ^1^College of Veterinary Medicine, Hunan Agricultural University, Changsha, China; ^2^Hunan Engineering Technology Research Center of Veterinary Drugs, Hunan Agricultural University, Changsha, China; ^3^Veterinary Drug Laboratory, Hunan Institute of Animal and Veterinary Science, Changsha, China; ^4^National Research Center of Engineering and Technology for Utilization of Botanical Functional Ingredients, Hunan Agricultural University, Changsha, China

**Keywords:** *Aeromonas hydrophila*, EnvZ/OmpR, growth, stress response, virulence

## Abstract

*Aeromonas hydrophila* is an important zoonotic pathogen responsible for septicemia, diarrhea and gastroenteritis, and has attracted considerable attention. The EnvZ/OmpR two-component system (TCS) mediates environmental stress responses in gram-negative bacteria. We investigated the role of the TCS in *A. hydrophila* by comparing the characteristics of the parental (*23-C-23*), EnvZ/OmpR knockout (*23-C-23:ΔEnvZ/OmpR*), and complemented strains (*23-C-23:CΔEnvZ/OmpR*). Under non-stress conditions, the *23-C-23:ΔEnvZ/OmpR* strain showed a significant decrease in growth rate compared to that of *23-C-23*. Transcriptome and metabonomic analysis indicated that many metabolic pathways were remarkably affected in the ΔEnvZ/OmpR strain, including the TCA cycle and arginine biosynthesis. In addition, the virulence of the ΔEnvZ/OmpR strain was attenuated in a Kunming mouse model. The ΔEnvZ/OmpR strain exhibited notably reduced tolerance to environmental stresses, including high temperature, different pH conditions, oxidative stress, and high osmotic stress. The downregulated expression of genes related to cell metabolism, motility, and virulence in the ΔEnvZ/OmpR mutant strain was further validated by real-time quantitative PCR. Consequently, our data suggest that the EnvZ/OmpR TCS is required for growth, motility, virulence, and stress response in *A. hydrophila*, which has significant implications in the development of novel antibacterial and vaccine therapies targeting EnvZ/OmpR against *A. hydrophila*.

## Introduction

*Aeromonas hydrophila*, an opportunistic aquatic pathogen that causes various diseases in both aquatic and terrestrial species, has attracted considerable attention owing to its high pathogenicity. It causes serious diseases in animals (Singh et al., 2011; Igbinosa et al., 2016; Liu et al., 2019) and humans (Zhang et al., 2012; Ventura et al., 2015), and is one of the most common pathogens posing serious public health problems. Survival and adaptation to hostile conditions can enhance and repress the expression of certain characteristics (Tsai et al., 1997; Boor, 2006; Mcmahon et al., 2007), promoting the survival of *A. hydrophila* in various environments. Environmental factors, such as temperature, nutrition, salinity, osmotic stress, pH, and glucose, affect *A. hydrophila* in a similar fashion (Pianetti et al., 2008; Jahid et al., 2013, 2015; Casabianca et al., 2015), making it difficult to prevent and control. Moreover, controlling disease outbreaks has become increasingly difficult owing to the emergence of drug-resistant strains of *A. hydrophila* (Vivekanandhan et al., 2002; Ma et al., 2018). Therefore, it is necessary to understand more about *A. hydrophila* to find novel and effective approaches for its control and treatment.

A two-component system (TCS) is a defense mechanism that bacteria have evolved in response to various environmental stresses. A TCS consists of a sensor kinase and response regulator for sensing environmental stress, transducing signals, and mediating gene expression, which allow bacteria to exhibit high survivability and proliferation under different environmental stresses as a key to their pathogenicity (Cai and Inouye, 2002; Krell et al., 2010). In recent years, the functions of some TCSs have been investigated. For example, in *Vibrio parahaemolyticus*, PhoR is responsible for bacterial motility and flagella assembly (Zhang et al., 2020b), and the Lux system is crucial for controlling bacterial biofilm formation (Liu et al., 2021b). In *Edwardsiella piscicida,* EsrA/EsrB is involved in the regulation of virulence gene expression (Yin et al., 2020a,b; Shao et al., 2021). Moreover, some other TCSs also play a crucial role in morphological differentiation (Li et al., 2020), antibiotic synthesis (Li et al., 2020), reactive oxygen species (ROS) resistance (Zhou et al., 2022), and multi-drug resistance (Guo et al., 2020).

The EnvZ/OmpR TCS is ubiquitous in gram-negative bacteria and regulates porin genes in response to changes in osmolarity, pH, and starvation (Cai and Inouye, 2002; Yuan et al., 2011; Chhabra et al., 2012; Chakraborty and Kenney, 2018; Kenney, 2019; Gerken et al., 2020; Kunkle et al., 2020). In *Escherichia coli*, silencing of the EnvZ/OmpR TCS affects the expression of more than 100 genes, leading to changes in a variety of bacterial physiological properties, such as chemotaxis, adhesion, biofilm formation, stress tolerance, and even pathogenicity (Oshima et al., 2002). The EnvZ/OmpR TCS also controls virulence in other pathogens, including *Shewanella oneidensis, Shigella flexneri*, *Yersinia pestis*, and *Vibrio cholerae* (Bernardini et al., 1990; Yuan et al., 2011; Reboul et al., 2014; Kunkle et al., 2020). In *A. hydrophila*, the EnvZ/OmpR two-component regulatory system has recently been reported to be involved in intracellular survival (Du et al., 2022). Interestingly, our previous study confirmed that the EnvZ/OmpR was the only dramatically upregulated TCS in the induction of *A. hydrophila WCX23* into colistin-resistant strain *23-C-23*, and contribute to colistin resistance of this bacteria by regulating the expression of various genes (Liu et al., 2021a). However, few other functions of the EnvZ/OmpR TCS in *A. hydrophila* have been described. Silencing *A. hydrophila* OmpR impairs bacterial virulence but does not represent EnvZ/OmpR TCS overall function (Zhang et al., 2020a). Therefore, studies on other functions of the EnvZ/OmpR TCS in *A. hydrophila* are warranted.

Since the EnvZ/OmpR TCS plays a key role in *A. hydrophila* resistance to colistin, a thorough understanding of the function of EnvZ/OmpR in this microbe is crucial for preventing, controlling, and treating *A. hydrophila* infections. In this work, the biological function of EnvZ/OmpR in colistin-resistant *A. hydrophila* was determined by comparing the parental and EnvZ/OmpR knockout strain. We demonstrated that the EnvZ/OmpR TCS contributes to cell growth, virulence, and stress response in *A. hydrophila*, which enhances our understanding of the EnvZ/OmpR TCS in the regulation of *A. hydrophila* pathogenicity and stress resistance.

## Materials and methods

### Strains, plasmids and growth conditions

The strains and plasmids used in this study were from our laboratory (Hunan Engineering Research Center of Veterinary Drug, Changsha, Hunan, China; Table 1). The mutant (*23-C-23:ΔEnvZ/OmpR*) and complemented mutant (*23-C-23:CΔEnvZ/OmpR*) were constructed in our previously published study (Liu et al., 2021a). The information regarding these strains has been described in our previous article. All strains were grown on tryptone soy agar (TSA; Oxoid, Basingstoke, United Kingdom) or cultured in tryptone soy broth (TSB; Oxoid, Basingstoke, United Kingdom) at 28°C. Colistin (Macklin, Shanghai, China) was used at the following concentrations: 4 mg/L for *23-C-23:ΔEnvZ/OmpR*, and 128 mg/L for *23-C-23* and *23-C-23:CΔEnvZ/OmpR*.

**Table 1:** Strains and plasmids used in this study.

Bacterial strains/plasmids	Description	Source
*23-C-23*	*WCX23* for 23 passages with colistin	Our laboratory
*23-C-23:ΔEnvZ/OmpR*	EnvZ/OmpR knockout from *23-C-23*	Our laboratory
*23-C-23:CΔEnvZ/OmpR*	EnvZ/OmpR complementation from *23-C-23:ΔEnvZ/OmpR*	Our laboratory
pUC57-Apr	Plasmid containing apramycin resistance gene apr	Liu et al., 2021a
pCVD442	A suicide plasmid	Liu et al., 2021a

### Growth curve

The overnight culture was adjusted with TSB liquid medium to an OD_600_ of 0.1, and then 200 μL of each bacterial suspension was dispensed into 96-well plates, and then the plates were incubated in an automatic microplate reader (BioTek, United States) at 28°C. The OD_600_ of each culture was real time monitored every 1 h for 16 h to detect the growth.

### Transcriptome analysis

In brief, the strains were cultured to log-phase OD_600_  = 0.6 ± 0.05) at 28°C, and then the cells were harvested and washed twice by sterilized PBS. Total RNA was extracted using Qiagen RNeasy Mini kits (Qiagen, Hilden, Germany), and quantified using a NanoDrop 2000 spectrophotometer (Thermo Fisher, Waltham, MA, United States). rRNA was depleted, and cDNA libraries were prepared as previously described (Liu et al., 2021a). The cDNA libraries were further sequenced by Illumina Hiseq 2000 system (Majorbio, Shanghai, China). Only genes with a log_2_ fold change > 1 (upregulation) or < −1 (downregulation), and a *p*-value < 0.05 were defined significantly differential expressed genes (DEGs). Gene Ontology (GO) and Kyoto Encyclopedia of Genes and Genomes (KEGG) enrichment pathway analysis of DEGs was performed using the R package.

### Quantitative real-time PCR analysis

Total RNA was extracted from bacteria in log-phase (OD_600_ = 0.6 ± 0.05) using a bacterial RNA kit (OMEGA, Norcross, Georgia, United States), and reverse transcribed using the Prime-Script® RT reagent kit (Takara, Tokyo, Japan). cDNA was amplified using a SYBR Premix Ex Taq II kit (Takara, Tokyo, Japan) in a qTOWER Real-Time System (Analytik Jena, Germany). All experiments were carried in triplicates. All data were normalized to 16S rRNA (internal reference gene) levels and analyzed using the 2^−ΔΔCT^ method. The transcript-specific primers used for quantitative real-time PCR (qRT-PCR) are listed in Supplementary Table 1.

### Metabolomic analysis

The strains were cultured to log-phase (OD_600_ = 0.6 ± 0.05) at 28°C, washed twice with precooled PBS buffer, and centrifuged at 10,000× *g* for 10 min at 4°C. After the supernatant was discarded, precooled methanol/acetonitrile/water (V/V, 2:2:1) was added and sonicated in an ice bath for 1 h. The mixture was incubated at −20°C for 1 h, centrifuged at 14,000× *g* at 4°C for 20 min, and then collected samples. The samples collected were analyzed using a UPLC-ESI-Q-Orbitrap-MS system (UHPLC, Shimadzu Nexera X2 LC-30 AD, Shimadzu, Japan) coupled with Q Exactive Plus (Thermo Scientific, San Jose, United States). Additionally, quality control samples were prepared and analyzed in the same way. The details of the method can be found in the Supplementary material. The differential metabolites were obtained using a statistically significant threshold of variable influence on projection (VIP) values obtained from the OPLS-DA model and two-tailed Student’s *t*-test (*p*-value) on the normalized raw data at univariate analysis level. Metabolites with VIP values > 1.0 and *p*-value < 0.05 were considered to be significantly differential metabolites. KEGG enrichment analyses were carried out with the Fisher’s exact test, and FDR correction for multiple testing was performed. Enriched KEGG pathways were nominally statistically significant at the *p* < 0.05 level.

### Virulence assay

Forty healthy Kunming mice (KM) weighing 20 ± 1 g (licensed: SCXK2019-0002), were bought from Hunan SJA Laboratory Animal Co. LTD (Hunan, China). The strains were cultured to log-phase (OD_600_  = 0.6 ± 0.05) at 28°C, and adjusted to an appropriate concentration. The mice were randomly divided into four groups of 10 individuals and injected intraperitoneally with 0.1 ml of each respective strain (*23-C-23*, *23-C-23:ΔEnvZ/OmpR,* and *23-C-23:CΔEnvZ/OmpR*) at doses of 1 × 10^7^ CFU. The other group injected 0.1 mL of sterile saline only. Mice were observed at 2 h intervals post-challenge, and clinical symptoms and mortality were recorded.

### Biofilm formation assay

The strains were cultured to log-phase (OD_600_ = 0.6 ± 0.05) at 28°C, and adjusted to an OD_600_ of 0.1, and 200 μl of culture (1:100 dilution) was dispensed into 96-well plates and incubated at 28°C. A 200 μl aliquot of fresh TSB was added to the plates as a blank control. After 24 h incubation, the medium was discarded, and the plates were washed three times with sterile PBS. The biofilm was fixed with 200 μl of methanol for 15 min and incubated with 200 μl of 1% crystal violet for 15 min. After several washes with ddH_2_O and air-dried, the dried biofilm was solubilized in 200 μL 95% ethanol for 10 min. The absorbance was measured at 595 nm.

### Motility assays

The strains were cultured to log-phase (OD_600_ = 0.6 ± 0.05) at 28°C. One microliter of bacterial suspension was spotted onto a 0.3% TSA plate, and incubated at 28°C for 24 h. The motility diameters of bacteria were measured.

### Chemotaxis assays

The strains were cultured to log-phase (OD_600_ = 0.6 ± 0.05) at 28°C. A capillary tube was closed at one end and filled with mucus, and the other end was dipped into the bacterial suspension. After 1 h, the liquid in the capillary was removed and double-diluted for counting. The number of bacteria in the capillary tube was measured.

### Temperature stress

The viability of strains was examined at 37 and 42°C to assess high-temperature tolerance of each strain. The strains were cultured to log-phase (OD_600_ = 0.6 ± 0.05) at 28°C. The culture was adjusted with TSB liquid medium to an OD_600_ of 0.1, then the bacterial suspension incubation at 28 (as a control), 37, and 42°C for 24 h. Then, the cultures were double-diluted with sterile PBS and coated onto TSA plates for counting. The ratio of colony-forming units (CFUs) in treatment group to control group was calculated to assess the percent survival.

### Acid and alkali stress

The strains were cultured to log-phase (OD_600_ = 0.6 ± 0.05) at 28°C. Bacterial suspension was collected, washed twice with sterile PBS, and treated in TSB at pH 5.0, pH 6.0, pH 7.0 (as a control), pH 8.0 or pH 9.0, at 28°C for 30 min. Next, viable cells were plated on TSA plates for counting after dilution. The ratio of CFUs in treatment group to that control group was calculated to assess the percent survival.

### Oxidative stress

The strains were cultured to log-phase (OD_600_ = 0.6 ± 0.05) at 28°C. Bacterial suspension was collected, washed twice with sterile PBS, and then suspended in PBS with 0.1 M H_2_O_2_, and without H_2_O_2_ as a control. After 20 min of incubation, viable cells were counted on TSA agar plates. The ratio of CFUs in treatment group to control group was calculated to assess the percent survival.

### Osmotic stress

The strains were cultured to log-phase (OD_600_ = 0.6 ± 0.05) at 28°C. The cells were collected, and resuspended in fresh TSB with 0.5 M NaCl, and adjusted to an OD_600_ of 0.1. 200 μl of each bacterial suspension was dispensed into 96-well plates, and then the plates were incubated in an automatic microplate reader at 28°C. The OD_600_ of each culture was real time monitored every 1 h for 16 h to detect the growth.

### Statistical analyses

All the experiments were tested at least three times. All data were expressed as the mean ± SD. Student’s *t*-test and analysis of variance (ANOVA) of GraphPad Prism 8 software were used to process and analyze routine data. *p* < 0.05 represents statistically significant.

## Results

### Envz/OmpR deletion reduces the growth of *23-C-23*

**Figure 1:**
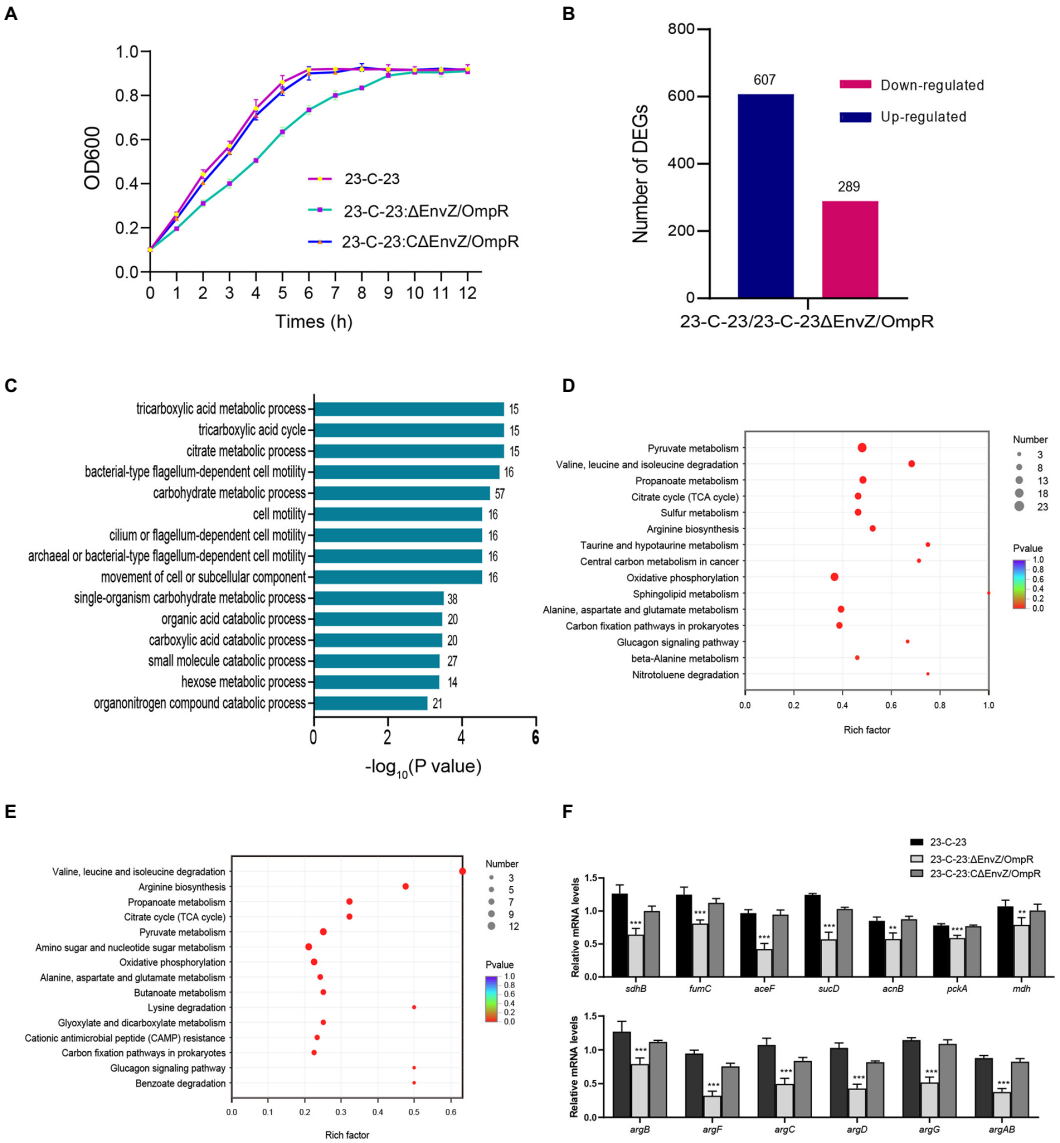
Effect of EnvZ/OmpR on growth of *23-C-23*. **(A)** Growth curve of *23-C-23*, *23-C-23:ΔEnvZ/OmpR,* and *23-C-23:CΔEnvZ/OmpR*. **(B)** Numbers of increased and decreased differentially expressed genes (DEGs). Genes with a log_2_ fold change > 1 or < −1, and a *p*-value < 0.05 were defined significantly DEGs. **(C)** GO biology processes. The y-axis represents GO terms, and the x-axis represents the cluster frequency of the DEGs. The number represents the number of genes enriched. **(D)** KEGG pathway enrichment analysis of all DEGs. **(E)** KEGG pathway enrichment analysis of significantly downregulated genes. The size of the dots indicates the number of expressed genes in the pathways, and the color of the dots represents the negative logarithm of value of p of the pathway. **(F)** The expression levels of metabolic-related genes (up: TCA cycle; down: arginine biosynthesis). Values are expressed as the mean ± SD of three individual experiments. ^**^p < 0.01, and ^***^p < 0.001 compared with control group.

Growth curve were initially examined in order to investigate whether envZ/ompR deletion strain affected the growth rate, compared to *23-C-23*. The results from the growth curves showed that the deletion of EnvZ/OmpR in *23-C-23* caused a significant decrease in the growth rate, but complementation with EnvZ/OmpR restored this rate (Figure 1A), suggesting that EnvZ/OmpR participated in the regulation of the growth of *A. hydrophila*. Transcriptome analysis showed that a total of 4,843 genes were annotated. With *p* < 0.05 and ∣log_2_Fold Change∣ > 1 as the threshold, a total of 896 differentially expressed genes (DEGs) were identified of which 607 were upregulated and 289 were downregulated (Figure 1B). Enrichment analysis revealed significant changes in 72 GO biological processes, of which the top 15 were involved in the tricarboxylic acid (TCA) cycle, carbohydrate metabolic process, and cell motility (Figure 1C). KEGG pathway analysis demonstrated that these DEGs were mainly associated with the TCA cycle, pyruvate metabolism, sulfur metabolism, arginine biosynthesis, and oxidative phosphorylation (Figure 1D). Interestingly, these downregulated DEGs were also involved in TCA cycle, pyruvate metabolism, arginine biosynthesis, and oxidative phosphorylation pathways (Figure 1E).

Based on these results, we further determined the expression levels of 13 representative metabolic genes using qRT-PCR (Figure 1F), which were selected from the KEGG pathways that were significantly enriched in relation to the TCA cycle and arginine biosynthesis. Specifically, we found that the mRNA expression of all genes in the TCA cycle and arginine biosynthesis was downregulated in *23-C-23:ΔEnvZ/OmpR* compared to that in the parent *23-C-23* strain, whereas there was no significant difference between that of *23-C-23:CΔEnvZ/OmpR* and *23-C-23*, which was consistent with the transcriptome analysis. These data imply that the EnvZ-OmpR-mediated growth rate decrease is associated with downregulated TCA cycle and arginine biosynthesis genes.

**Figure 2:**
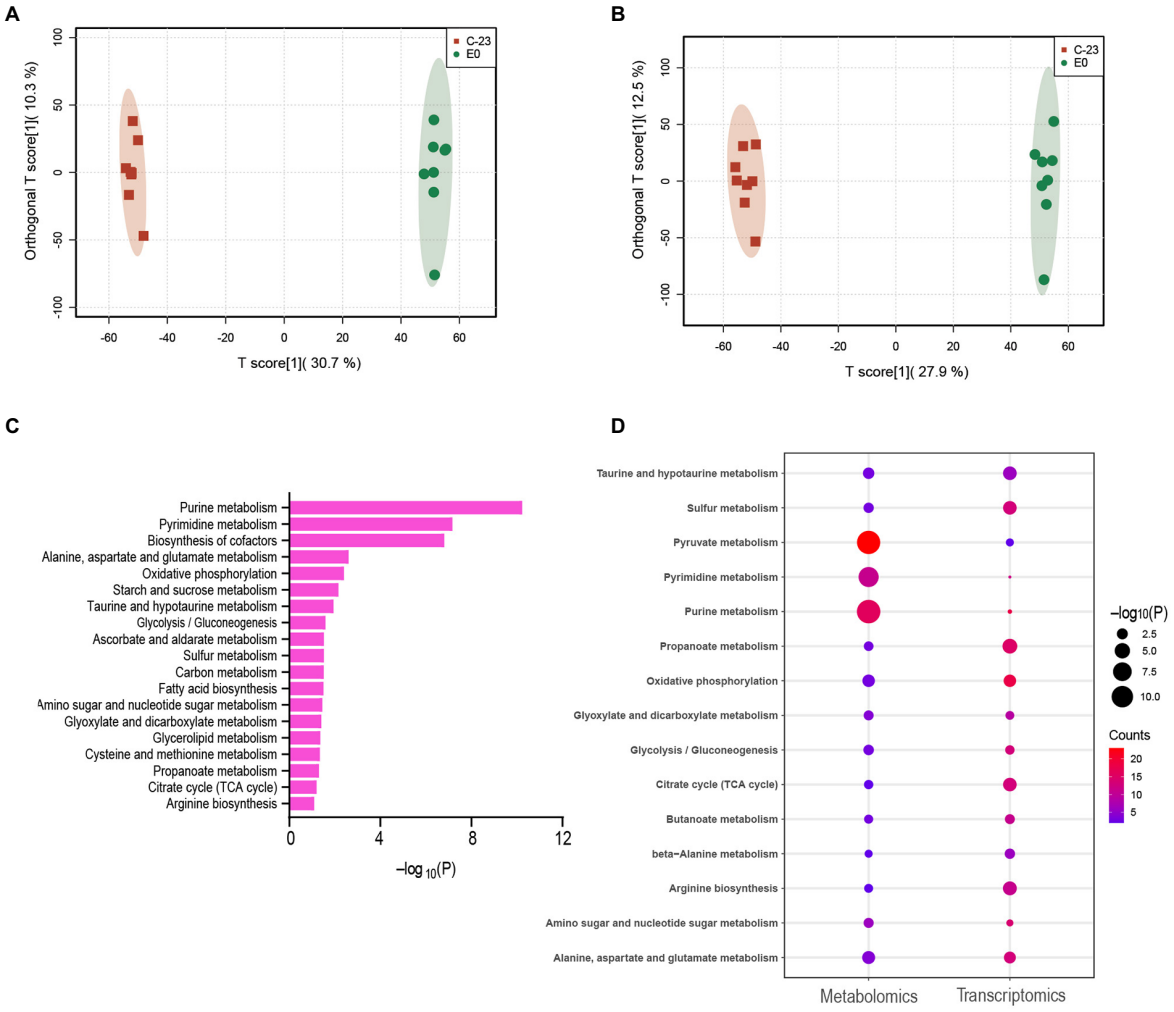
Comparative metabolomics profiles of *23-C-23* and *23-C-23:ΔEnvZ/OmpR*. **(A,B)** Orthogonal partial least squares discriminant analysis (OPLS-DA) of positive and negative ionization dataset for the *23-C-23* and EnvZ/OmpR mutant strains. (**A**: positive mode; **B**: negative mode), Each dot represents a biological replicate of each strain. Red square represents *23-C-23*, green dot represents *23-C-23:ΔEnvZ/OmpR*. **(C)** KEGG pathway enrichment analysis of differential abundant metabolites. The y-axis represents KEGG terms, and the x-axis represents negative logarithm of value of *p.* **(D)** Metabolomics and transcriptomics share significant KEGG bubble charts. The size of the dots indicates the number of expressed genes or metabolites in the pathways, and the color of the dots represents the negative logarithm of value of *p* of the pathway.

In addition, comparative metabolomics revealed the regulatory role of the EnvZ-OmpR on the metabolism of *A. hydrophila*. According to the metabolomic profiles, the OPLS-DA score separated *23-C-23* and ΔEnvZ/OmpR, implying that *23-C-23* and ΔEnvZ/OmpR were not similar (Figures 2A,B). With vip > 1.0 and *p* < 0.05 as the threshold for significance, a total of 402 significantly differential metabolites, including 253 increased and 149 decreased (Supplementary Figure 1). KEGG pathway enrichment analysis (Figure 2C) showed that the knockout of the EnvZ/OmpR TCS specifically affected carbohydrate, amino acid, and nucleotide metabolism, which emphasizes the transcriptomic data. The common significant pathways enriched in transcriptome and metabolome were analyzed (Figure 2D). In addition, the PPI network was mapped using combined transcriptome and metabolome analysis (Supplementary Figure 2). Taken together, these data suggest that the EnvZ/OmpR TCS affects the growth rate of *A. hydrophila* by regulating the metabolic processes of bacteria.

### EnvZ/OmpR deletion impairs virulence of *23-C-23*

**Figure 3:**
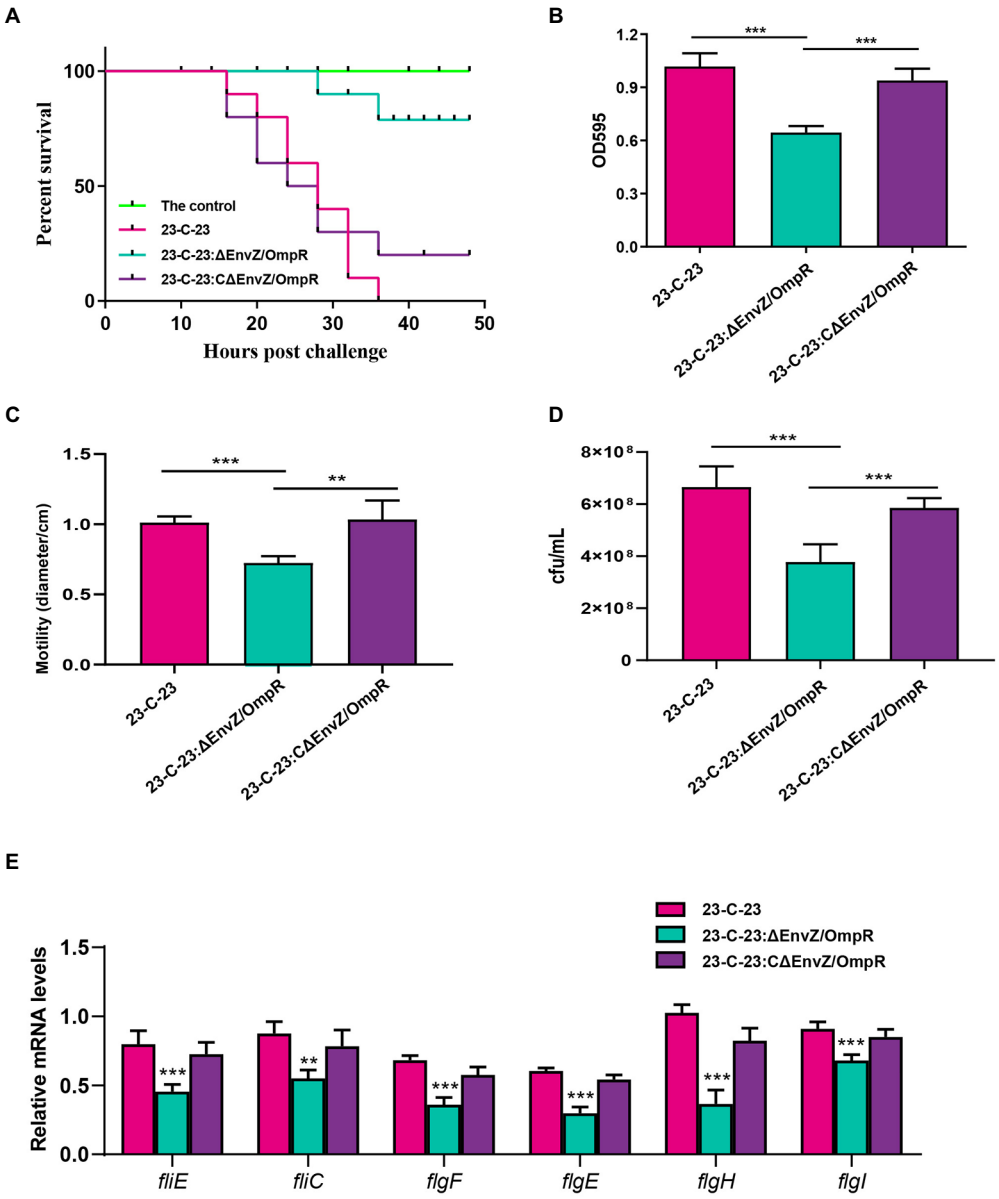
Effect of EnvZ/OmpR on the virulence of *23-C-23*. **(A)** Survival curves for KM mouse in experiment infection. The mice was injected intraperitoneally with 0.1 ml of each respective strain (*23-C-23*, *23-C-23,ΔEnvZ/OmpR,* and *23-C-23,CΔEnvZ/OmpR*) at doses of 1 × 10^7^ CFU. The mortality of mice was recorded. **(B)** Motility assay. The strains were cultured to log-phase. One microliter of bacterial suspension was spotted onto a 0.3% TSA plate, and incubated at 28°C for 24 h. The motility diameters of bacteria were measured. **(C)** Chemotaxis assay. The strains were cultured to log-phase. A capillary tube was closed at one end and filled with mucus, and the other end was dipped into the bacterial suspension. After 1 h, the liquid in the capillary was removed for counting. The number of bacteria in the capillary tube was measured. **(D)** Biofilm formation ability of *23-C-23*, *23-C-23:ΔEnvZ/OmpR,* and *23-C-23:CΔEnvZ/OmpR*. **(E)** The expression levels of flagellar assembly-related genes. Values are expressed as the mean ± SD of three individual experiments. ^**^p < 0.01, and ^***^p < 0.001 compared with *23-C-23*.

Mice in the *23-C-23* group showed clinical signs, including chills, shivering, hair bristling, and eye closure 2 h post-challenge, with fatalities occurring 16 h post-challenge, thereby leading to a survival rate of 0%. Mice in the *23-C-23:CΔEnvZ/OmpR* group showed clinical signs 2 h post-challenge, with fatalities occurring 16 h post-challenge, leading to a survival rate of 20%. Mice in the *23-C-23:ΔEnvZ/OmpR* group showed clinical signs at 14 h post-challenge, with fatalities occurring at 28 h post-challenge, which led to a survival rate of 80%. The control mice showed no clinical effects, and had a 100% survival rate (Figure 3A). These data suggest that EnvZ/OmpR is involved in the regulation of *A. hydrophila* virulence.

To further confirm the mechanism of EnvZ/OmpR in the regulation of the virulence, several biological characteristics of wild-type strain, *23-C-23: ΔenvZ/ompR* and *23-C-23:CΔenvZ/ompR* were compared. As shown in Figure 3B, biofilm-forming ability was significantly reduced after knockout of EnvZ/OmpR and restored after complementation, suggesting that EnvZ/OmpR contributes to biofilm formation by *A. hydrophila*. In addition, compared with *23-C-23* and *23-C-23:CΔEnvZ/OmpR*, the bacterial motility of the EnvZ/OmpR deletion strain decreased by 28.2% (Figure 3C), and bacterial chemotaxis decreased by 43.3% (Figure 3D).

Furthermore, deletion of EnvZ/OmpR significantly decreased the expression levels of flagellar assembly-related genes in contrast to *23-C-23* (Figure 3E), which was consistent with the transcriptomics. When EnvZ/OmpR was complemented, the expression of these genes recovered. Biofilm formation, motility, and chemotaxis are important bacterial virulence factors. Taken together, these data suggest that EnvZ/OmpR reduces bacterial motility, chemotaxis, and biofilm formation by downregulating the expression of genes related to bacterial flagellar assembly, thereby weakening the virulence of the *23-C-23:ΔEnvZ/OmpR* strain.

### EnvZ/OmpR deletion diminishes stress tolerance of *23-C-23*

**Figure 4:**
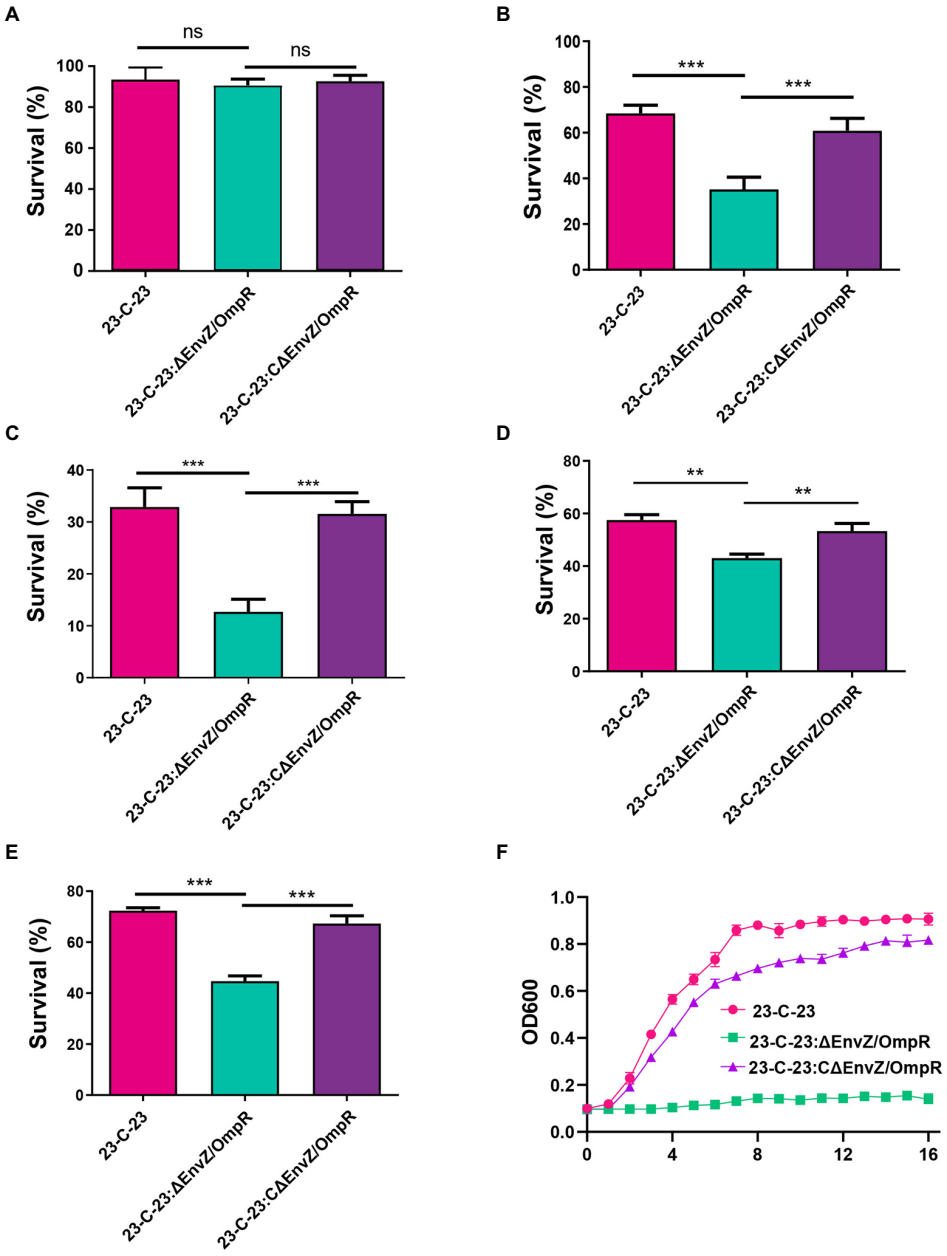
Effects of EnvZ/OmpR on the viability of *23-C-23* under different stress conditions. **(A,B)** Temperature stress assay. The strains were cultured to log-phase. The culture was adjusted with TSB liquid medium to an OD_600_ of 0.1, then the bacterial suspension incubation at 28 (as a control), 37, and 42°C for 24 h. Then the cultures were double-diluted with sterile PBS and coated onto TSA plates. **(A)** 37°C, **(B)** 42°C. **(C,D)** Acid and alkali tolerance. The strains were cultured to log-phase. Bacteria were treated for 30 min in TSB at different PH (pH 5.0, pH 7.0, and pH 9.0). Next, viable cells were plated on TSA plates after dilution. The ratio of CFUs in treatment group to that control group was calculated to assess the percent survival. **(C)** pH 5.0, **(D)** pH 9.0. **(E)** Oxidative stress assay. The strains were cultured to log-phase. Bacteria were treated for 20 min in PBS with 0.1 M H_2_O_2_, and without H_2_O_2_ as a control. Viable cells were counted on TSA agar plates. The ratio of CFUs in treatment group to control group was calculated to assess the percent survival. **(F)** Osmotic stress assay. The cells were collected, and resuspended in fresh TSB with 0.5 M NaCl to an OD_600_ of 0.1. The OD_600_ of each culture was real time monitored every 1 h for 16 h to detect the growth. Values are expressed as the mean ± SD of three individual experiments. ^**^p < 0.01, and ^***^p < 0.001.

To explore the effect of EnvZ/ompR on the stress tolerance of *A. hydrophila*, we compared the growth characteristics of *23-C-23*, *23-C-23: ΔenvZ/ompR,* and *23-C-23:CΔenvZ/ompR* strains under different stress conditions. When incubated at 37°C, there was no significant difference between the survival rates of wild-type and EnvZ/OmpR deletion bacteria (Figure 4A). However, a significant difference was observed at 42°C (Figure 4B). Based on this result we speculate that the existence of a critical temperature signal value for activating the EnvZ/OmpR TCS. Lower survival and tolerance of *23-C-23:ΔEnvZ/OmpR*, compared to *23-C-23*, were observed at acidic and alkaline pH (Figures 4C,D). In contrast, survival of *23-C-23:CΔEnvZ/OmpR* was restored. In addition, we found that *23-C-23:ΔenvZ/ompR* were less tolerant to acidic pH than alkaline pH. Compared with *23-C-23*, the survival of EnvZ/OmpR deletion strains was reduced by 62.5% and 42.1% at pH = 5 (Figure 4C) and pH = 6 (Supplementary Figure 3A), respectively, and by 21% and 27.1% at pH = 8 (Figure 4D) and pH = 9 (Supplementary Figure 3B), respectively. After treatment with 0.5 M H_2_O_2_, a lower survival rate was observed for the *23-C-23:ΔEnvZ/OmpR* strain than for *23-C-23*. In all, 55% of the *23-C-23:ΔEnvZ/OmpR* cells were killed, whereas 72% and 67% of the *23-C-23* and *23-C-23:CΔEnvZ/OmpR* cells survived, respectively (Figure 4E). The osmotic stress analysis revealed that *23-C-23:ΔEnvZ/OmpR* was more sensitive to NaCl challenge than *23-C-23* and *23-C-23:CΔEnvZ/OmpR* strains and did not grow under high osmotic pressure (Figure 4F). As expected, complementation with EnvZ/OmpR in *23-C-23:ΔEnvZ/OmpR* restored osmotic stress resistance. Taken together, these results reveal that the expression of the EnvZ/OmpR TCS contributes to the growth of *A. hydrophila* in various harsh environments.

## Discussion

Although colistin is the last resort for clinical treatment of multi-drug resistant Gram-negative bacteria infection, the continuous emergence of colistin-resistant bacteria posed a serious threat to the efficacy of antibiotic therapy (Laplante et al., 2018; Huang et al., 2021; Jang et al., 2021). In addition, bacteria can be adapted to adverse and detrimental conditions through stress-triggered regulatory systems (Yin et al., 2021; Dawan and Ahn, 2022), such as TCS, which has increased the difficulty of prevention and control of pathogenic bacteria. Numerous studies have identified EnvZ/OmpR functioning as a factor in bacterial survival and development in response to various harsh environments (Chakraborty and Kenney, 2018; Gerken et al., 2020; Kunkle et al., 2020). Our previous study confirmed that the EnvZ/OmpR TCS is closely associated with *A. hydrophila* resistance to colistin (Liu et al., 2021a). In the present study, we found that ΔEnvZ/OmpR significantly reduced the growth rate, virulence, and stress response of colistin-resistant *A. hydrophila*, suggesting that the EnvZ/OmpR TCS is not only involved in *A. hydrophila* resistance to colistin, but also regulates its growth, virulence, and stress tolerance. These results are noteworthy, as the EnvZ/OmpR TCS could be a vital target for the prevention, control, and treatment of colistin-resistant *A. hydrophila*.

First, we observed that deletion of EnvZ/OmpR dramatically reduced the growth rate of *23-C-23* even under non-stress conditions, and this result agrees with previous reports on *E. coli* (Oshima et al., 2002), but contrasts with *S. oneidensis* (Yuan et al., 2011), which suggests that the function of EnvZ/OmpR TCS is not always the same in diverse bacterial species. The EnvZ/OmpR TCS, as a global regulator, may directly and/or indirectly participate in other important growth-related pathways. To further understand the regulatory effects of the EnvZ/OmpR TCS, we compared the transcriptomic and metabolomic characteristics of the ΔEnvZ/OmpR mutant and wild-type strains. We determined that EnvZ/OmpR depletion in *23-C-23* cells seriously disrupted several important metabolic pathways, especially the TCA cycle and arginine metabolism. The TCA cycle and arginine biosynthesis which are important carbon and energy metabolism pathways are the main sources of energy acquisition for bacterial survival. qRT-PCR analysis further confirmed this observation, in which the expression levels of the genes involved in these metabolic processes were significantly decreased in ΔEnvZ/OmpR. These data suggest that deletion of EnvZ/OmpR in *A. hydrophila* limits its nutrient uptake through downregulation of genes in major metabolic pathways, thereby reducing energy availability and leading to slower growth. Although the results are interesting, we still do not elucidate the mechanism by which EnvZ/OmpR regulates bacterial metabolism. In order to explore the mechanisms by which EnvZ/OmpR regulates bacterial metabolism, it is necessary to construct deletion mutants of metabolic genes and test their association, which is the focus of our further work.

In this study, the ΔEnvZ/OmpR strain showed a long lethality time and low lethality in mice, suggesting that EnvZ/OmpR deletion impairs the virulence of *A. hydrophila*. Studies have shown that motility and chemotaxis are important features of virulence in many pathogens (Tomas, 2012). The significantly reduced bacterial motility and chemotaxis in ΔEnvZ/OmpR compared to the restored ability in the complemented strain. This may be because the EnvZ/OmpR deletion downregulates flagellar assembly protein genes. It has been demonstrated that flagella confer locomotion and are often associated with virulence of most bacterial pathogens (Luo et al., 2016; Zhu et al., 2019, 2021). Our results indicate that EnvZ/OmpR mediates cell motility and virulence by controlling the expression of flagellar assembly genes. Moreover, previous studies have suggested that the fastest growing cells are significantly more virulent than those grown more slowly because they can rapidly reach pathogenic concentrations in the host (Marsh et al., 1994; Tipton and Rather, 2017). Bacteria typically use metabolic signals to regulate their metabolic and virulence functions. Therefore, we speculate that the reduced growth caused by the EnvZ/OmpR deletion may also be responsible for the diminished virulence of *A. hydrophila*.

During infection, *A. hydrophila* strains invade blood circulation through the wound or gastrointestinal tract. Consequently, they must adapt to extreme and rapidly changing environmental conditions, such as high temperature, extreme pH, osmotic pressure, and oxidative stress. Our results on growth characteristics of the ΔEnvZ/OmpR mutant under various stress conditions confirmed that the ΔEnvZ/OmpR mutants displayed dramatically defects in survival under extreme stress conditions, such as high temperature, low pH, high pH, high osmolarity, and oxidative stress, compared to *23-C-23*. The diminished stress tolerance of the ΔEnvZ/OmpR mutant strain may be due to the lack of EnvZ/OmpR TCS in *A. hydrophila*, which weakens the reception, transmission, and response to complex environmental changes. In addition, biofilm formation is thought to be a common mechanism that contributed to bacteria survive in stressful environments (Gao et al., 2019; Masmoudi et al., 2019). We confirmed that EnvZ/OmpR deletion diminishes biofilm formation by *A. hydrophila*, which suggests that the EnvZ/OmpR TCS regulates biofilm formation in response to environmental changes. Other studies have suggested that silencing or knocking out the EnvZ/OmpR TCS reduces biofilm formation (Lin et al., 2018; Shi et al., 2018; Zhang et al., 2020a). Given the attenuated tolerance of the ΔEnvZ/OmpR mutant strain to diverse environmental stresses, the possibility of survival of such mutant cells in the host is very low, which may be the main reason for the diminished virulence of *A. hydrophila*.

In conclusion, in this study, we confirmed that the EnvZ/OmpR TCS contributes to *A. hydrophila* cell growth, virulence, biofilm formation, and environmental stress tolerance, suggesting that the EnvZ/OmpR TCS is essential for *A. hydrophila* survival, reproduction, and disease outbreaks. Hence, to further screen inhibitors targeting EnvZ/OmpR TCS as potential colistin adjuvant may be a new idea for the development of novel antibacterial therapies to prevent and control colistin-resistant *A. hydrophila*. However, our study was not able to clarify the mechanism by which EnvZ/OmpR regulates bacterial metabolism, and the effect of changed metabolism on virulence and stress tolerance in colistin-resistant *A. hydrophila*. Therefore, our future work will focus on the study of the action mechanism of EnvZ/OmpR TCS.

## Data availability statement

The datasets presented in this study can be found in online repositories. The names of the repository/repositories and accession number(s) can be found at: https://www.ncbi.nlm.nih.gov/, PRJNA706457 and https://www.ebi.ac.uk/metabolights/, MTBLS5805.

## Ethics statement

The animal study was reviewed and approved by the Animal Ethics Committee of Hunan Agricultural University.

## Author contributions

ZS, GX, JuL, and XZ designed the study. GX performed the main experiments and wrote the manuscript. YY, JiL, JY, and NX helped to collect, analyze, and interpret the data. YY, JY, and NX helped to revise the manuscript. All authors contributed to the article and approved the submitted version.

## Funding

This research was supported by the Hunan Provincial Natural Science Foundation of China (2021JJ40234).

## Conflict of interest

The authors declare that the research was conducted in the absence of any commercial or financial relationships that could be construed as a potential conflict of interest.

## Publisher’s note

All claims expressed in this article are solely those of the authors and do not necessarily represent those of their affiliated organizations, or those of the publisher, the editors and the reviewers. Any product that may be evaluated in this article, or claim that may be made by its manufacturer, is not guaranteed or endorsed by the publisher.

## References

[ref1] Bernardini M. L. Fontaine A. Sansonetti P. J. (1990). The two-component regulatory system ompR-envZ controls the virulence of *Shigella flexneri*. J. Bacteriol. 172, 6274–6281. doi: 10.1128/jb.172.11.6274-6281.1990, PMID: 2121709 PMC526810

[ref2] Boor K. J. (2006). Bacterial stress responses: what doesn't kill them can make then stronger. PLoS Biol. 4:e23. doi: 10.1371/journal.pbio.0040023, PMID: 16535775 PMC1326283

[ref3] Cai S. J. Inouye M. (2002). EnvZ-OmpR interaction and osmoregulation in *Escherichia coli*. J. Biol. Chem. 277, 24155–24161. doi: 10.1074/jbc.M110715200, PMID: 11973328

[ref4] Casabianca A. Orlandi C. Barbieri F. Sabatini L. Di Cesare A. Sisti D.. (2015). Effect of starvation on survival and virulence expression of *Aeromonas hydrophila* from different sources. Arch. Microbiol. 197, 431–438. doi: 10.1007/s00203-014-1074-z, PMID: 25533849

[ref5] Chakraborty S. Kenney L. J. (2018). A new role of OmpR in acid and osmotic stress in *salmonella* and *E. coli*. Front. Microbiol. 9:2656. doi: 10.3389/fmicb.2018.02656, PMID: 30524381 PMC6262077

[ref6] Chhabra G. Upadhyaya T. Dixit A. (2012). Molecular cloning, sequence analysis and structure modeling of OmpR, the response regulator of *Aeromonas hydrophila*. Mol. Biol. Rep. 39, 41–50. doi: 10.1007/s11033-011-0708-3, PMID: 21533905

[ref7] Dawan J. Ahn J. (2022). Bacterial stress responses as potential targets in overcoming antibiotic resistance. Microorganisms 10: 1385. doi: 10.3390/microorganisms10071385, PMID: 35889104 PMC9322497

[ref8] Du Z. Zhang M. Qin Y. Zhao L. Huang L. Xu X.. (2022). The role and mechanisms of the two-component system EnvZ/OmpR on the intracellular survival of *Aeromonas hydrophila*. J. Fish Dis., 1–13. doi: 10.1111/jfd.13684, PMID: 35822274

[ref9] Gao T. Ding M. Yang C. H. Fan H. Chai Y. Li Y. (2019). The phosphotransferase system gene ptsH plays an important role in MnSOD production, biofilm formation, swarming motility, and root colonization in *Bacillus cereus* 905. Res. Microbiol. 170, 86–96. doi: 10.1016/j.resmic.2018.10.002, PMID: 30395927

[ref10] Gerken H. Vuong P. Soparkar K. Misra R. (2020). Roles of the EnvZ/OmpR two-component system and Porins in iron Acquisition in *Escherichia coli*. mBio 11, e01192–20. doi: 10.1128/mBio.01192-20 32576675 PMC7315122

[ref11] Guo Q. Dong L. Wang P. Su Z. Liu X. Zhao W.. (2020). Using a phenotype microarray and transcriptome analysis to elucidate multi-drug resistance regulated by the PhoR/PhoP two-component system in *Bacillus subtilis* strain NCD-2. Microbiol. Res. 239:126557. doi: 10.1016/j.micres.2020.126557, PMID: 32688186

[ref12] Huang S. Wang S. Li Y. Fang M. Kou Z. Chen B.. (2021). Prevalence and transmission of mobilized colistin resistance (mcr-1) gene positive *Escherichia coli* in healthy rural residents in Shandong province. China. Microbiol Res 253:126881. doi: 10.1016/j.micres.2021.126881, PMID: 34592562

[ref13] Igbinosa I. H. Igbinosa E. O. Okoh A. I. (2016). Antibiogram characterization and putative virulence genes in *Aeromonas* species isolated from pig fecal samples. Environ. Sci. Pollut. Res. Int. 23, 12199–12205. doi: 10.1007/s11356-016-6421-y, PMID: 26971520

[ref14] Jahid I. K. Lee N. Y. Kim A. Ha S. D. (2013). Influence of glucose concentrations on biofilm formation, motility, exoprotease production, and quorum sensing in *Aeromonas hydrophila*. J. Food Prot. 76, 239–247. doi: 10.4315/0362-028X.JFP-12-321, PMID: 23433371

[ref15] Jahid I. K. Mizan M. F. Ha A. J. Ha S. D. (2015). Effect of salinity and incubation time of planktonic cells on biofilm formation, motility, exoprotease production, and quorum sensing of *Aeromonas hydrophila*. Food Microbiol. 49, 142–151. doi: 10.1016/j.fm.2015.01.016, PMID: 25846924

[ref16] Jang H. Choi S. Y. Mun W. Jeong S. H. Mitchell R. J. (2021). Predation of colistin- and carbapenem-resistant bacterial pathogenic populations and their antibiotic resistance genes in simulated microgravity. Microbiol. Res. 255:126941. doi: 10.1016/j.micres.2021.126941 34915266

[ref17] Kenney L. J. (2019). The role of acid stress in salmonella pathogenesis. Curr. Opin. Microbiol. 47, 45–51. doi: 10.1016/j.mib.2018.11.006 30529007

[ref18] Krell T. Lacal J. Busch A. Silva-Jimenez H. Guazzaroni M. E. Ramos J. L. (2010). Bacterial sensor kinases: diversity in the recognition of environmental signals. Annu. Rev. Microbiol. 64, 539–559. doi: 10.1146/annurev.micro.112408.134054, PMID: 20825354

[ref19] Kunkle D. E. Bina X. R. Bina J. E. (2020). *Vibrio cholerae* OmpR contributes to virulence repression and fitness at alkaline pH. Infect. Immun. 88, e00141–20. doi: 10.1128/IAI.00141-20, PMID: 32284367 PMC7240085

[ref20] Laplante K. Cusumano J. Tillotson G. (2018). Colistin for the treatment of multidrug-resistant infections. Lancet Infect. Dis. 18, 1174–1175. doi: 10.1016/S1473-3099(18)30611-X 30507395

[ref21] Li L. Zhao Y. Ma J. Tao H. Zheng G. Chen J.. (2020). The orphan histidine kinase PdtaS-p regulates both morphological differentiation and antibiotic biosynthesis together with the orphan response regulator PdtaR-p in *Streptomyces*. Microbiol. Res. 233:126411. doi: 10.1016/j.micres.2020.126411, PMID: 31981905

[ref22] Lin T. H. Chen Y. Kuo J. T. Lai Y. C. Wu C. C. Huang C. F.. (2018). Phosphorylated OmpR is required for type 3 fimbriae expression in *Klebsiella pneumoniae* under hypertonic conditions. Front. Microbiol. 9:2405. doi: 10.3389/fmicb.2018.02405, PMID: 30369914 PMC6194325

[ref23] Liu J. Xiao G. Zhou W. Yang J. Wang Y. Wu Y.. (2021a). Various novel Colistin resistance mechanisms interact to facilitate adaptation of *Aeromonas hydrophila* to complex Colistin environments. Antimicrob. Agents Chemother. 65:e0007121. doi: 10.1128/AAC.00071-21, PMID: 33903105 PMC8373241

[ref24] Liu J. Xie L. Zhao D. Yang T. Hu Y. Sun Z.. (2019). A fatal diarrhoea outbreak in farm-raised *Deinagkistrodon acutus* in China is newly linked to potentially zoonotic *Aeromonas hydrophila*. Transbound. Emerg. Dis. 66, 287–298. doi: 10.1111/tbed.13020, PMID: 30222905

[ref25] Liu M. Zhu X. Zhang C. Zhao Z. (2021b). LuxQ-LuxU-LuxO pathway regulates biofilm formation by *Vibrio parahaemolyticus*. Microbiol. Res. 250:126791. doi: 10.1016/j.micres.2021.126791, PMID: 34090181

[ref26] Luo G. Huang L. Su Y. Qin Y. Xu X. Zhao L.. (2016). flrA, flrB and flrC regulate adhesion by controlling the expression of critical virulence genes in *vibrio alginolyticus*. Emerg. Microbes Infect. 5:e85. doi: 10.1038/emi.2016.82 27485498 PMC5034100

[ref27] Ma S. Sun C. Hulth A. Li J. Nilsson L. E. Zhou Y.. (2018). Mobile colistin resistance gene mcr-5 in porcine *Aeromonas hydrophila*. J. Antimicrob. Chemother. 73, 1777–1780. doi: 10.1093/jac/dky110, PMID: 29659855 PMC6005042

[ref28] Marsh P. D. Mcdermid A. S. Mckee A. S. Baskerville A. (1994). The effect of growth rate and haemin on the virulence and proteolytic activity of Porphyromonas gingivalis W50. Microbiology 140, 861–865. doi: 10.1099/00221287-140-4-861, PMID: 8012602

[ref29] Masmoudi F. Abdelmalek N. Tounsi S. Dunlap C. A. Trigui M. (2019). Abiotic stress resistance, plant growth promotion and antifungal potential of halotolerant bacteria from a Tunisian solar saltern. Microbiol. Res. 229:126331. doi: 10.1016/j.micres.2019.126331, PMID: 31521945

[ref30] Mcmahon M. A. Xu J. Moore J. E. Blair I. S. Mcdowell D. A. (2007). Environmental stress and antibiotic resistance in food-related pathogens. Appl. Environ. Microbiol. 73, 211–217. doi: 10.1128/AEM.00578-06, PMID: 17142359 PMC1797128

[ref31] Oshima T. Aiba H. Masuda Y. Kanaya S. Sugiura M. Wanner B. L.. (2002). Transcriptome analysis of all two-component regulatory system mutants of *Escherichia coli* K-12. Mol. Microbiol. 46, 281–291. doi: 10.1046/j.1365-2958.2002.03170.x, PMID: 12366850

[ref32] Pianetti A. Manti A. Boi P. Citterio B. Sabatini L. Papa S.. (2008). Determination of viability of *Aeromonas hydrophila* in increasing concentrations of sodium chloride at different temperatures by flow cytometry and plate count technique. Int. J. Food Microbiol. 127, 252–260. doi: 10.1016/j.ijfoodmicro.2008.07.024, PMID: 18765166

[ref33] Reboul A. Lemaitre N. Titecat M. Merchez M. Deloison G. Ricard I.. (2014). *Yersinia pestis* requires the 2-component regulatory system OmpR-EnvZ to resist innate immunity during the early and late stages of plague. J Infect Dis 210, 1367–1375. doi: 10.1093/infdis/jiu274, PMID: 24813471

[ref34] Shao S. Li C. Zhao L. Zhang Y. Yin K. Wang Q. (2021). Interplay between ferric uptake regulator fur and horizontally acquired virulence regulator EsrB coordinates virulence gene expression in *Edwardsiella piscicida*. Microbiol. Res. 253:126892. doi: 10.1016/j.micres.2021.126892, PMID: 34673373

[ref35] Shi C. Li M. Muhammad I. Ma X. Chang Y. Li R.. (2018). Combination of berberine and ciprofloxacin reduces multi-resistant *salmonella* strain biofilm formation by depressing mRNA expressions of luxS, rpoE, and ompR. J. Vet. Sci. 19, 808–816. doi: 10.4142/jvs.2018.19.6.808, PMID: 30304890 PMC6265579

[ref36] Singh B. R. Gulati B. R. Virmani N. Chauhan M. (2011). Outbreak of abortions and infertility in thoroughbred mares associated with waterborne *Aeromonas hydrophila*. Indian J. Microbiol. 51, 212–216. doi: 10.1007/s12088-011-0088-3, PMID: 22654167 PMC3209884

[ref37] Tipton K. A. Rather P. N. (2017). An OmpR-EnvZ two-component system Ortholog regulates phase variation, osmotic tolerance, motility, and virulence in *Acinetobacter baumannii* strain AB5075. J. Bacteriol. 199, e00705–16. doi: 10.1128/JB.00705-16, PMID: 27872182 PMC5237114

[ref38] Tomas J. M. (2012). The main *Aeromonas* pathogenic factors. ISRN Microbiol. 2012:256261. doi: 10.5402/2012/256261 23724321 PMC3658858

[ref39] Tsai G. J. Tsai F. C. Kong Z. L. (1997). Effects of temperature, medium composition, pH, salt and dissolved oxygen on haemolysin and cytotoxin production by *Aeromonas hydrophila* isolated from oyster. Int. J. Food Microbiol. 38, 111–116. doi: 10.1016/S0168-1605(97)00094-9, PMID: 9506276

[ref40] Ventura R. J. Muhi E. De Los Reyes V. C. Sucaldito M. N. Tayag E. (2015). A community-based gastroenteritis outbreak after typhoon Haiyan, Leyte, Philippines, 2013. Western Pac. Surveill. Response J. 6, 1–6. doi: 10.5365/wpsar.2014.5.1.010, PMID: 25960917 PMC4410107

[ref41] Vivekanandhan G. Savithamani K. Hatha A. A. Lakshmanaperumalsamy P. (2002). Antibiotic resistance of *Aeromonas hydrophila* isolated from marketed fish and prawn of South India. Int. J. Food Microbiol. 76, 165–168. doi: 10.1016/S0168-1605(02)00009-0, PMID: 12038573

[ref42] Yin K. Peng Y. Ahmed M. A. H. Ma J. Xu R. Zhang Y.. (2020a). PepA binds to and negatively regulates esrB to control virulence in the fish pathogen *Edwardsiella piscicida*. Microbiol. Res. 232:126349. doi: 10.1016/j.micres.2019.126349, PMID: 31816594

[ref43] Yin K. Zhang J. Ma J. Jin P. Ma Y. Zhang Y.. (2020b). MviN mediates the regulation of environmental osmotic pressure on esrB to control the virulence in the marine fish pathogen *Edwardsiella piscicida*. Microbiol. Res. 239:126528. doi: 10.1016/j.micres.2020.126528, PMID: 32622286

[ref44] Yin W. L. Zhang N. Xu H. Gong X. X. Long H. Ren W.. (2021). Stress adaptation and virulence in *vibrio alginolyticus* is mediated by two (p)ppGpp synthetase genes, relA and spoT. Microbiol. Res. 253:126883. doi: 10.1016/j.micres.2021.126883, PMID: 34626929

[ref45] Yuan J. Wei B. Shi M. Gao H. (2011). Functional assessment of EnvZ/OmpR two-component system in *Shewanella oneidensis*. PLoS One 6:e23701. doi: 10.1371/journal.pone.0023701, PMID: 21886811 PMC3160321

[ref46] Zhang M. Kang J. Wu B. Qin Y. Huang L. Zhao L.. (2020a). Comparative transcriptome and phenotype analysis revealed the role and mechanism of ompR in the virulence of fish pathogenic *Aeromonas hydrophila*. Microbiology 9:e1041. doi: 10.1002/mbo3.1041 PMC7349151 32282134

[ref47] Zhang Y. Liu H. Gu D. Lu X. Zhou X. Xia X. (2020b). Transcriptomic analysis of PhoR reveals its role in regulation of swarming motility and T3SS expression in *Vibrio parahaemolyticus*. Microbiol. Res. 235:126448. doi: 10.1016/j.micres.2020.126448, PMID: 32114363

[ref48] Zhang Q. Shi G. Q. Tang G. P. Zou Z. T. Yao G. H. Zeng G. (2012). A foodborne outbreak of *Aeromonas hydrophila* in a college, Xingyi City, Guizhou, China, 2012. Western Pac. Surveill. Response J. 3, 39–43. doi: 10.5365/wpsar.2012.3.4.018, PMID: 23908938 PMC3729099

[ref49] Zhou M. Huang Y. Zhang Y. Wang Q. Ma Y. Shao S. (2022). Roles of virulence regulator ToxR in viable but non-culturable formation by controlling reactive oxygen species resistance in pathogen *vibrio alginolyticus*. Microbiol. Res. 254:126900. doi: 10.1016/j.micres.2021.126900, PMID: 34700184

[ref50] Zhu S. Schniederberend M. Zhitnitsky D. Jain R. Galan J. E. Kazmierczak B. I.. (2019). *In situ* structures of polar and lateral flagella revealed by Cryo-electron tomography. J. Bacteriol. 201, e00117–19. doi: 10.1128/JB.00117-19, PMID: 31010901 PMC6560136

[ref51] Zhu D. Wang S. Sun X. (2021). FliW and CsrA govern Flagellin (FliC) synthesis and play pleiotropic roles in virulence and physiology of *Clostridioides difficile* R20291. Front. Microbiol. 12:735616. doi: 10.3389/fmicb.2021.735616, PMID: 34675903 PMC8523840

