# Supplementary material for: Contribution of the EnvZ/OmpR two-component system to growth, virulence and stress tolerance of colistin-resistant Aeromonas hydrophila
Source: Front Microbiol. 2022 Oct 12;13:1032969. doi: 10.3389/fmicb.2022.1032969 (PMC9597241; doi:10.3389/fmicb.2022.1032969)
Supplement: Supplementary file 1 [file Data_Sheet_1.pdf]

Supplementary Material

1 Metabonomics analysis

1.1 Sample preparation

The strains were grown to log-phase (about $OD_{600} = 0.6$), washed twice with precooled PBS buffer and centrifuged at 10000 g for 10 min at 4 °C. The metabolites were extracted from cell residue with 1 mL precooled methanol/acetonitrile/water (v/v, 2:2:1) under sonication for 1 h in ice baths. The mixture was incubated at -20 °C for 1 h followed by centrifugation at 14,000 g, 4 °C for 20 min, and then the samples collected were analyzed using a UPLC-ESI-Q-Orbitrap-MS system (UHPLC, Shimadzu Nexera X2 LC-30AD, Shimadzu, Japan) coupled with Q-Exactive Plus (Thermo Scientific, San Jose, USA). Additionally, to ensure data quality for metabolic profiling, Quality control (QC) samples were prepared by pooling aliquots of all samples that were representative of the all samples under analysis, and used for data normalization. QC samples were prepared and analyzed with the same procedure as that for the experiment samples in each batch. Dried extracts were then dissolved in 50% acetonitrile. Each sample was filtered with a disposable 0.22 μm cellulose acetate and transferred into 2 mL HPLC vials for LC-MS analysis.

1.2 UHPLC-MS/MS analysis

Metabolomics profiling was analyzed using a UPLC-ESI-Q-Orbitrap-MS system (UHPLC, Shimadzu Nexera X2 LC-30AD, Shimadzu, Japan) coupled with Q-Exactive Plus (Thermo Scientific, San Jose, USA). The flow rate was 0.3 mL/min and the mobile phase contained: A: 25 mM ammonium acetate and water and B: 100% acetonitrile. The gradient was 95% B for 1 min and was linearly reduced to 65% in 7 min, and then reduced to 35% in 2 min and maintained for 0.5 min, and then increased to 95% in 0.5 min, with 2 min re-equilibration period employed. Both electrospray ionization (ESI) positive-mode and negative mode were applied for MS data acquisition. The HESI source conditions were set as follows: Spray Voltage:3.8kv (+) and 3.2kv (-); Capillary Temperature:320 (±); Sheath Gas:30 (±); Aux Gas:5 (±); Probe Heater Temp:350 (±); S-Lens RF Level:50. In MS only acquisition, the instrument was set to acquire over the m/z range 80-1200. The full MS scans were acquired at a resolution of 70,000 @m/z 200, and 17,500 @m/z 200 for MS/MS scan. The maximum injection time was set to for 100 ms for MS and 50 ms for MS/MS. The isolation window for MS2 was set to 2 m/z and the normalized collision energy (stepped) was set as 10, 20 and 30 for fragmentation.

1.3 Data preprocessing and filtering

The raw MS data were processed using MS-DIAL for peak alignment, retention time correction and peak area extraction. The metabolites were identified by accuracy mass (mass tolerance < 0.01Da) and MS/MS data (mass tolerance < 0.02Da) which were matched with HMDB, MassBank and other public databases and our self-built

metabolite standard library. In the extracted-ion features, only the variables having more than 50% of the nonzero measurement values in at least one group were kept.

1.4 Multivariate statistical analysis

R(version:4.0.3) and R packages was used for all multivariate data analyses and modeling. Data were mean-centered using Pareto scaling. Models were built on principal component analysis (PCA) and orthogonal partial least-square discriminant analysis (OPLS-DA). All the models evaluated were tested for over fitting with methods of permutation tests. The descriptive performance of the models was determined by R2X (cumulative) (perfect model: R2X (cum) = 1) and R2Y (cumulative) (perfect model: R2Y (cum) = 1) values while their prediction performance was measured by Q2 (cumulative) (perfect model: Q2 (cum) = 1) and a permutation test (n = 200). The permuted model should not be able to predict classes: R2 and Q2 values at the Y-axis intercept must be lower than those of Q2 and the R2 of the non-permuted model. OPLS-DA allowed the determination of discriminating metabolites using the variable importance on projection (VIP). The VIP score value indicates the contribution of a variable to the discrimination between all the classes of samples. The mean VIP value is 1, and usually VIP values over 1 are considered as significant. A high score in agreement with a strong discriminatory ability and thus constitutes a criterion for the selection of biomarkers.

The discriminating metabolites were obtained using a statistically significant threshold of variable influence on projection (VIP) values obtained from the OPLS-DA model and two-tailed Student's t test (p value) on the normalized raw data at univariate analysis level. The p value was calculated by one-way analysis of variance (ANOVA) for multiple groups analysis. Metabolites with VIP >1.0 and p value < 0.05 were considered to be statistically significant metabolites. Fold change was calculated as the logarithm of the average mass response (area) ratio between two arbitrary classes. On the other side, the identified differential metabolites were used to perform cluster analyses with R package.

2 Supplementary Tables

Supplementary Table 1. Primer sequences for qRT-PCR

Gene	Primer F (5'-3')	Primer R (5'-3')
16s RNA	GAGATACGGGAGTGCCTTCG	GTGCTGGCAACAAAGGACAG
sdhB	CAGTGGGAGAAGGTCAAAC	TACACTCGTACAGGCCAT
fumC	AACCTCAACGAGGTGCT	ACTGGCTCTTGTTCACCT
aceF	ACCGATGAGGTGGACTT	GTCAAACCAGGCGTTCAT
sucD	GACCTATGAAGCGGTCAAG	TCTCCAGGATGTCGATGAA
acnB	TACTGCGAGCCCAAGAT	AGGACTGCATGGTCAGAT
pckA	CACGCCAACAAGGTGAT	CAGACAGGAAGTGGTACTTG
mdh	TCGTTCCGACCTGTTCA	GACCGGATTGGTGATGATG

argB	CAAGACCTTTCTGGACGAAC	CTTCTTGGTGGAGGTGAGA
argF	GGGTTTGACGTGGCTTATAC	GCCTGGTTGATCTGATAAGG
argC	CCTCACCTTGGCAACTAT	TCGTAGGCCTTGAGGTA
argD	TGCCTACTCCGATGGTT	GCAGGTATTGTCGGAGATG
argG	GTCTATGAGGGCACCTATCT	TGCAACCGTGGGAAATG
argAB	AGATCGTGCGGGAAAGT	CACCAGAATACCTTCCTCCT
fliE	AGAGATGCAGGCTCTCAA	TCGTTGACGTTGCCAAG
fliC	GGGCTATGTTGGTTCTTTCC	CCACAGTAGCTTTGCCTTTC
flgF	CAAGGATGGCCAGAATGAA	CTGACGTTGCAACCGAATA
flgE	TCCAGCTAACCCGATCAA	ACATCACCTAGACGGATCTC
flgH	CAGGTCAACGGCATCTATTC	TCTGAGTGTTGGCCTTCT
flgI	CTGCTGCAAAGCTTCCT	GGGTATTGATCACCACCTTG

3 Supplementary Figures

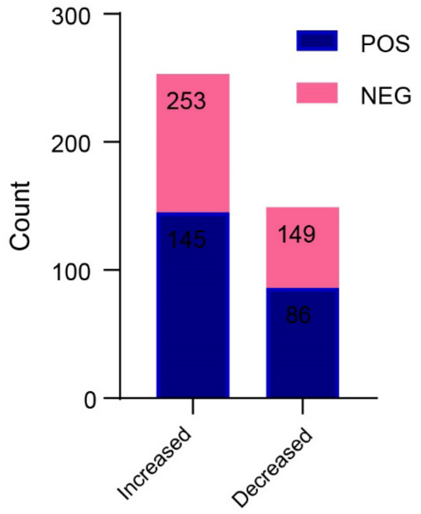

Supplementary Figure 1. Number of differential abundant metabolites increased and decreased. Metabolites with vip > 1.0 and p < 0 .05 were defined significantly differential metabolites.

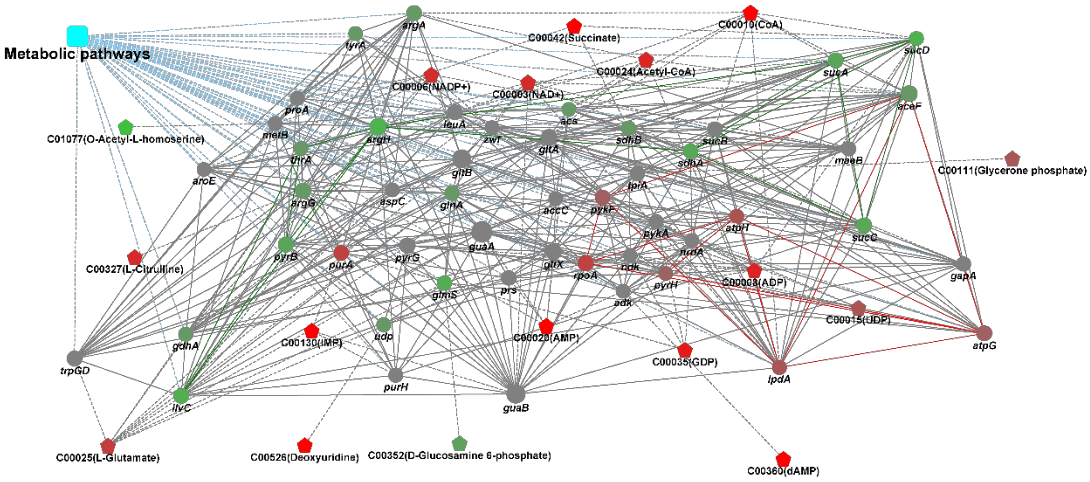

Supplementary Figure 2. PPI network of combined metabolome and transcriptome analysis. The circular nodes in the diagram represent genes and the pentagon represent metabolites. Green in the graph indicates downregulation, red indicates upregulation, and gray indicates no significant change.

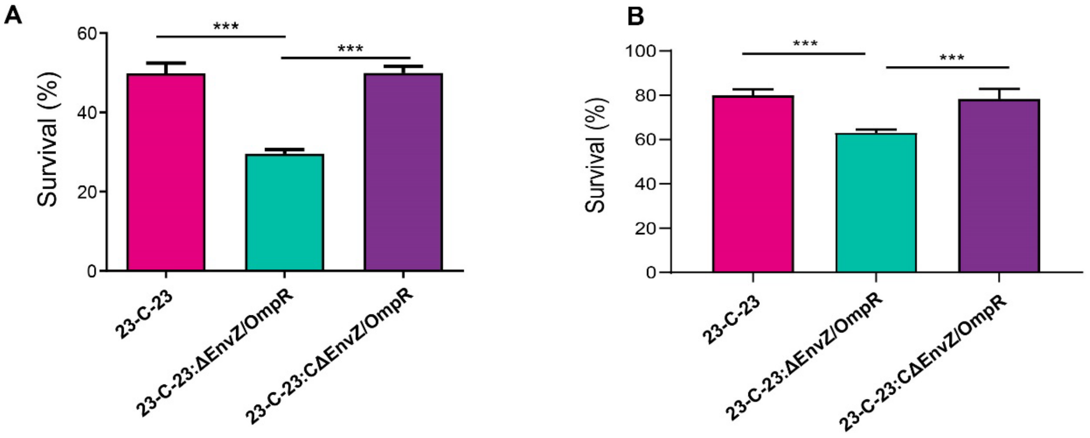

Supplementary Figure 3. Effects of EnvZ/OmpR on the viability of *A. hydrophila 23-C-23* under Acidic and alkaline conditions. The strains were cultured to log-phase. Bacteria were treated for 30 min in TSB at different PH (pH 6.0, pH 7.0 and pH 8.0). Next, viable cells were plated on TSA plates after dilution. The ratio of CFUs in treatment group to that control group was calculated to assess the percent survival. A. pH=6.0. B. pH=8.0.
